# The emergence of COVID-19 over-concern immediately after the cancelation of the measures adopted by the dynamic zero-COVID policy in China

**DOI:** 10.3389/fpubh.2023.1319906

**Published:** 2024-01-05

**Authors:** Fengyi Hao, Zhisong Zhang, Sam S. S. Lau, Soon-Kiat Chiang, Dewen Zhou, Wanqiu Tan, Xiangdong Tang, Roger Ho

**Affiliations:** ^1^Sleep Medicine Center, Mental Health Center, Translational Neuroscience Center, West China Hospital, Sichuan University, Chengdu, China; ^2^Faculty of Education, Huaibei Normal University, Huaibei, China; ^3^Research Centre for Environment and Human Health, School of Continuing Education, Hong Kong Baptist University, Kowloon, Hong Kong SAR, China; ^4^Multidisciplinary Research Centre, School of Continuing Education, Hong Kong Baptist University, Kowloon, Hong Kong SAR, China; ^5^College of International Education, School of Continuing Education, Hong Kong Baptist University, Kowloon, Hong Kong SAR, China; ^6^Institute of Bioresource and Agriculture, Hong Kong Baptist University, Kowloon, Hong Kong SAR, China; ^7^Department of Psychological Medicine, Yong Loo Lin School of Medicine, National University of Singapore, Singapore, Singapore; ^8^Institute of Psychology, Chinese Academy of Sciences, Beijing, China; ^9^National University of Singapore (Chongqing) Research Institute, Chongqing, China; ^10^Institute for Health Innovation and Technology (iHealthtech), National University of Singapore, Singapore, Singapore

**Keywords:** COVID-19, overconcern, China, dynamic zero-COVID, illness anxiety, depression, anxiety, stress

## Abstract

**Background:**

This study aimed to report the prevalence of COVID-19 over-concern and its associated factors after the relaxation of the health-protective measures in China.

**Methods:**

A team of seven experts in psychiatry and psychology specializing in COVID-19 mental health research from China, Hong Kong, and overseas reached a consensus on the diagnostic criteria for COVID-19 over-concern. Individuals had to meet at least five of the following criteria: (1) at least five physical symptoms; (2) stocking up at least five items related to protecting oneself during the COVID-19 pandemic; (3) obsessive-compulsive symptoms related to the COVID-19 pandemic; (4) illness anxiety related to the COVID-19 pandemic; (5) post-traumatic stress symptoms; (6) depression; (7) anxiety; (8) stress and (9) insomnia. An online survey using snowball sampling collected data on demographics, medical history, views on COVID-19 policies, and symptoms of COVID-19 over-concern. Multivariate linear regression was performed using significant variables from the previous regressions as independent variables against the presence of COVID-19 over-concern as the dependent variable. Breush-Pagan test was used to assess each regression model for heteroskedasticity of residuals.

**Results:**

1,332 respondents from 31 regions in China participated in the study for 2 weeks from December 25 to 27, 2022, after major changes in the zero-COVID policy. After canceling measures associated with the dynamic zero-COVID policy, 21.2% of respondents fulfilled the diagnostic criteria for COVID-19 over-concern. Factors significantly associated with COVID-19 over-concern were poor self-rated health status (*β* = 0.07, *p* < 0.001), concerns about family members getting COVID-19 (*β* = 0.06, *p* < 0.001), perceived usefulness of COVID-19 vaccine (*β* = 0.03, *p* = 0.012), impact on incomes, employment and studies (*β* = 0.045, *p* < 0.001) and impact on families (*β* = 0.03, *p* = 0.01).

**Conclusion:**

After removing measures associated with the dynamic zero-COVID policy in China, approximately one-fifth of respondents met the diagnostic criteria for COVID-19 over-concern.

## Introduction

In December 2021, China implemented the dynamic zero-COVID policy to contain localized COVID-19 flare-ups through timely interventions (1). This policy entailed a series of stringent control measures, including (i) medical isolation of close contacts of confirmed cases, (ii) massive nucleic acid testing, (iii) citywide home quarantine, (iv) electronic health pass to gain entry to public places, (v) travel restrictions, and (vi) quarantine on arrival for citizens and foreigners from other countries and enforcement of protective measures (e.g., wearing face masks in public areas) (2). While the policy kept the number of COVID-19 cases low by cutting off transmissions in the communities, it exerted downward pressure on the economy (2) and led to personal burnout (3). A significant turning point in this policy occurred on December 7, 2022, when China’s National Health Commission announced the complete removal of the dynamic zero-COVID policy, which was immediately put into effect (4). This shift signaled the removal of certain measures that had become an integral part of daily life, signifying a new phase in China’s battle against the pandemic.

After the relaxation of the above measures, there had been a rapid and substantial increase in the number of COVID-19 cases in China. Local news reported that specific abnormal illness behaviors occurred, including physical symptoms (e.g., sore throat, difficulty in breathing), hypochondriac thought of having COVID-19 infection despite the negative result, the compulsion to repeat COVID-19 testing and stocking of medications, depression, anxiety, insomnia and post-traumatic stress symptoms (5). Notably, the media’s role in amplifying negative information during a pandemic, as Bagus et al. (6) highlighted, could contribute to mass over-concern among the population (6). While previous studies focused on COVID-19 panic under restrictive measures (7, 8), some scholars have observed an increase in COVID-19 over-concern when the Zero-COVID Policy is lifted (9). Considering the rapid transmission rate of COVID-19 and the typical recovery period for most individuals, there is a limited timeframe for conducting this study. Hence, it is crucial for the research team to investigate the prevalence of COVID-19 over-concern during this critical period, and our findings will serve as a historical reference.

This study was initiated and completed shortly after the policy changes, and at that time, diagnostic criteria or recommendations for COVID-19 over-concern had not yet been established. The study team which comprises academic and clinical experts from China, Hong Kong and Singapore with experience in COVID-19 research reached a consensus and defined the diagnostic criteria for COVID-19 over-concern. The seven experts include two clinical psychiatrists from China with experience in COVID-19 mental health research, one professor of psychiatry from China with experience in COVID-19 research, two academic psychologists from China with experience in COVID-19 mental health research, one academic researcher from Hong Kong with experience in COVID-19 mental health research and one professor of psychiatry outside China who specialized in COVID-19 research and served as an external advisor.

The diagnostic criteria are based on the chain mediation model, which shows that the perceived impact of the pandemic was sequential mediators between physical symptoms resembling COVID-19 infection (i.e., the predictor) and consequent mental health status (i.e., the outcome) (10). The previous questionnaires on the psychological impacts of the COVID-19 pandemic and zero-COVID policy in China were also reviewed (3, 11–14). A person must fulfill at least five out of nine criteria, including (1) at least five physical symptoms; (2) stocking up at least five items related to protecting oneself during the COVID-19 pandemic; (3) obsessive-compulsive symptoms related to the COVID-19 pandemic; (4) illness anxiety related to the COVID-19 pandemic; (5) post-traumatic stress symptoms; (6) depression; (7) anxiety; (8) stress and (9) insomnia. The primary aim of this study was to establish the prevalence of COVID-19 over-concern in China shortly after major changes to the dynamic zero-COVID policy. The secondary aim was to identify the associations between demographics, health status, COVID-19-related information, views toward dynamic zero-COVI policy and severity of an individual with the defined symptoms of COVID-19 over-concern.

The study team had three hypotheses: (1) The COVID-19 over-concern was a common condition, and the prevalence would be higher than 10% when the dynamic zero-COVID measures were lifted; (2) Higher severity of physical symptoms would be associated with higher severity of psychological symptoms, including obsessive-compulsive symptoms, illness anxiety, depression, anxiety, stress, PTSD symptoms and insomnia; (3) The severity of COVID-19 over-concern among individuals was positively associated with the extent to which they perceive various aspects of their lives being affected.

The results of this study can assist health authorities develop a series of measures to help the public effectively manage different stages of a pandemic, particularly when strict policies are suddenly canceled.

## Materials and methods

### Setting and participants

The cross-sectional study used an anonymous online survey to assess the presence and severity of COVID-19 over-concern symptoms and views toward the zero-COVID-19 policy. We adopted a snowball sampling strategy focusing on recruiting the general public living in mainland China. Snowball sampling was a recruitment strategy commonly used during the COVID-19 pandemic, in which existing research participants were asked to identify other potential research participants (15). A sample size of 385 would give a sample size sufficient to draw assumptions of the population size of China at 95% confidence level and 5% margin of error. Data collection took place over 3 days (from 25 to 27 December 2022) after major changes in the zero-COVID policy for 2 weeks.

The inclusion criteria included: (1) at least 18 years of age; (2) residing in China at the time of the survey; and (3) being able to read and understand simplified Chinese, a written language used in Mainland China. The exclusion criteria included: (1) known to be positive for COVID-19 infection at the time of the survey; and (2) currently being hospitalized due to COVID-19 infection at the time of the survey; (3) history of severe mental illnesses (e.g., schizophrenia, intellectual disability, dementia) that affect the capacity to provide informed consent and fill the questionnaires. People who were known to be positive for COVID-19 infection and hospitalized due to COVID-19 infection at the time of the survey were encouraged not to participate in this study.

To achieve good quality control of responses, the following measures were applied: (1) Only one questionnaire could be submitted from one IP address to avoid duplicated entries from a single IP address or participant; (2) Use reCAPTCHA acknowledgement to avoid completing the questionnaire by computers or artificial intelligence systems; (3) If a participant submitted the questionnaire within 420 s, the questionnaire would be classified as invalid; (4) If a participant reported the highest level of education as a bachelor, master or PhD degree but the reported ages were less than 18 years, 21 years and 23 years, respectively, the questionnaire would be classified as invalid because a Chinese citizen could not enter the universities to pursue the above degrees below the minimum entry age set by the government (i.e., 18 years for bachelor, 21 years of master and 23 years for PhD degrees); (5) If a participant reported as married but the reported age was younger than 20 years for female and 22 years for male participants, the questionnaire would be classified as invalid because a Chinese citizen could not get married below the legal age of marriage set by the government (i.e., 20 years for women and 22 years for men).

### Procedure

Information about this study was posted on a dedicated website. All respondents provided informed consent before participation. The participants completed the online questionnaire through an online survey platform (‘SurveyStar,’ Changsha Ranxing Science and Technology, Shanghai, China) that could only reach participants who stayed in Mainland China (excluding Hong Kong and Macau Special Administrative Regions). The platform allowed each IP address to submit one questionnaire to avoid multiple submissions from the same participant. Expedited ethics approval was obtained from the Institutional Review Board (IRB) of the Huaibei Normal University, China (HBS-FDX-2022-012). The design of this study conformed to the principles embodied in the Declaration of Helsinki.

### Survey development

The previous questionnaire on the psychological impacts of the COVID-19 pandemic and zero-COVID policy in China was reviewed (3, 11–14). The experts of the study team reached a consensus that COVID-19 over-concern was defined and characterized by obsessive and compulsive symptoms in thinking and checking for COVID-19 symptoms and related information, having an illness anxiety of contracting COVID-19 infection, having anxiety, depression, stress or post-traumatic stress symptoms due to strict measures associated with the dynamic zero-COVID policy. The experts from the authors’ team selected questionnaires that were previously validated in Chinese and included additional questions related to the dynamic zero-COVID policy and the current situation in China. The structured online survey consisted of questions that covered the following areas: (1) demographics; (2) physical symptoms and health status in the past 14 days; (3) past COVID-19 history and strategies to cope with the COVID-19 pandemic; (4) views toward the measures associated with the dynamic zero-COVID policy; (5) the impact of dynamic zero-COVID policy; (6) the illness attitude scale with specific reference to COVID-19 infection; (7) Yale-Brown Obsessive Compulsive scale with specific reference to measures related to the dynamic zero-COVID policy; (8) Depression, Anxiety and Stress scale −21 items (DASS-21), (9) Impact of Event Scale–Revised (IES-R) and (10) Insomnia Severity Index (ISI).

Sociodemographic data, including age, gender, education level, marital status, living arrangement, occupation, and preferred place to work or study (e.g., home or office) were collected. Physical symptoms resembling COVID-19 infection in the past 14 days included chills, cough, coryza, difficulty breathing, dizziness, fever, headache, loss of taste and smell, myalgia, sore throat or other physical symptoms. Participants were asked to rate their physical health status and state any history of chronic medical illness. Past COVID-19 history includes the number of times contracting COVID-19 infection, vaccination history against COVID-19 infection, hoarding of the items to combat COVID-19 infection (e.g., antipyretics, alcohol swabs, facemasks, thermometers, traditional Chinese medicine to treat COVID-19 infection), last admission to Fangcang hospitals, a kind of large-scale, temporary, mobile hospitals built and used during the COVID-19 pandemic in China, number of times being lockdown, number of deaths due to COVID-19 infection in participants’ families and concerns of family members contracting COVID-19 infection.

The participants rated their views toward procedures adopted by the dynamic zero-COVID policy on a Likert scale: (i) massive nucleic acid testing; (ii) citywide home quarantine; (iii) booster of COVID-19 vaccination; (iv) electronic health pass; (v) travel restrictions; (vi) working or attending classes from home; (vii) limitation on the sales of antipyretics and other medications to treat influenza over the counter, (viii) closure of public places (e.g., swimming pool, karaoke, etc.), (ix) restriction to dine at food and beverage establishments, (x) wearing facemasks at indoor and outdoor venues. The participants also rated the impact of the COVID-19 pandemic and dynamic-zero COVID policy on a Likert scale: (i) on their lives; (ii) their incomes; (iii) families; and (iv) traveling.

### Psychiatric scales

The illness attitude scale (IAS) measured the illness anxiety associated with COVID-19 infection, a 29-item scale that has nine subscales including (1) worry about COVID-19 infection, (2) concerns about the discomfort associated with COVID-19 infection, (3) health habits, (4) hypochondriacal beliefs, (5) fear of death, (6) phobia of COVID-19 infection, (7) bodily preoccupations, (8) treatment experience, and (9) effects of COVID-19 symptoms (16). The total score is between 0 and 108, with a cut-off score of 44 and above to classify as a case of illness anxiety for COVID-19 infection (17). The Cronbach’s alpha reliability for the Chinese version of the IAS ranged from 0.68–0.82 for the four subscales (Patho-thanatophobia: 0.82; Symptom effect: 0.82; Treatment seeking: 0.74; Hypochondriacal belief: 0.68) (18). The test–retest reliability was 0.95 (18). For this study, the Cronbach’s alpha reliability for IAS is 0.91.

The Yale-Brown Obsessive Compulsive Scale Mandarin Chinese version (Y-BOCS) has 10 questions (19). The first half of the scale assesses common obsessions: excessive fears of contamination and recurring doubts about danger. The second half of the questionnaire assesses compulsions that help to lessen feelings of anxiety or other discomfort. The Chinese version of the Y-BOCS demonstrated excellent psychometric properties, including high internal consistency (0.88) and a cut-off score of 16 and above to classify a case of being obsessive-compulsive (20). For this study, the Cronbach’s alpha reliability of Y-BOCS is 0.83.

The post-traumatic symptoms associated with the COVID-19 pandemic and measures related to the zero-COVID policy were measured using the Impact of Event Scale-Revised (IES-R). The IES-R is a self-administered questionnaire validated in the Chinese population for determining the extent of post-traumatic stress in a public health crisis within 1 week of exposure to the COVID-19 pandemic in various cultures (21–26). The IES-R has been well-validated and extensively used for determining the extent of psychological impact in response to the COVID-19 pandemic among Chinese (27). The Cronbach’s alpha reliability for the Chinese version of IES-R was 0.949 (10). For this study, the Cronbach’s alpha reliability of IES-R is 0.96. The IES-R has three subscales measuring the avoidance, hyperarousal and intrusion (28). A total IESR score of 33 or over indicates the likely presence of post-traumatic stress disorder (28).

Depression, anxiety and stress associated with the cancelation of measures associated with the dynamic zero-COVID policy was measured by using the Depression, anxiety and stress scale (21-item) DASS-21 scale. The DASS-21 is a reliable and valid measure in assessing mental health in the Chinese (29), Filipino (30), Iranian (26), Polish (15), Spanish (23) and Vietnamese (31) during the COVID-19 pandemic. The cut-off score for the DASS-21 depression scale was ≥14; the DASS-21 anxiety scale was ≥10; andthe DASS-21 stress scale score was ≥19 (11). Wang et al. reported that the test–retest reliability over a 6-month interval was 0.39 to 0.46 for each of the 3 subscales of DASS-21 and 0.46 for the total DASS-21 score. Moderate convergent validity of the Depression and Anxiety subscales of DASS-21 demonstrated significant correlations with the Chinese Beck Depression Inventory and the Chinese State–Trait Anxiety Inventory (32). For this study, the Cronbach’s alpha reliability of DASS-21 is 0.96. The Cronbach’s alpha reliability of depression, anxiety and stress subscales are 0.9, 0.89 and 0.9, respectively.

Sleep quality was assessed by using the Insomnia Severity Index (ISI). The ISI assesses the severity of sleep difficulties, the extent to which sleep problems interfere with daily functioning and the impact of sleep problems on quality of life. The ISI was validated in Chinese during the COVID-19 pandemic (33). The cut-off score for clinical insomnia is greater or equal to 15.The Cronbach’s alpha reliability of the Chinese version ISI was 0.83 and the 2-week test–retest reliability was 0.79 (34). For this study, the Cronbach’s alpha reliability of ISI is 0.92.

For the entire survey, the Cronbach’s alpha reliability, Split-half reliability, Content Validity Index, and Kappa values were 0.967, 0.783, 0.967, and 0.967, respectively, reflecting excellent internal consistency, good reliability, and strong content validity. The Content Validity Index and Kappa values were evaluated by seven external experts who were not members of the study team, including two psychologists, four psychiatrists, and one epidemiologist (see Supplementary Table S1).

### Statistical analysis

Descriptive statistics were calculated for sociodemographic characteristics, physical health status, views toward measures adopted by the dynamic zero-COVID policy, and the perceived impact of the dynamic zero-COVID policy. Percentages of responses were calculated according to the number of respondents per response with respect to the number of total responses to a question. The total scores of the Y-BOCS, IAS, IES-R, DASS-21 and ISI. Their respective subscales were expressed as mean and standard deviation. The number and percentage of respondents above the cut-off scores for different scales, respondents reporting 5 or more COVID-19-related symptoms and hoarding 5 or more COVID-19-related precautionary items, were calculated. Linear regression was used to calculate the univariate associations between sociodemographic characteristics, physical health status, views toward measures adopted by the dynamic zero-COVID policy, and the perceived impact of the dynamic zero-COVID policy, against the total scores of Y-BOCS, illness attitude scale, IES-R, DASS-21, and ISI. The Variance Inflation Factor (VIF) was used to detect multicollinearity among the predictor variables. If the VIF is more than 5, the regression analysis is said to be highly correlated. Multivariate linear regression was then performed using significant variables from the previous regressions as independent variables, against presence of COVID-19 over-concern as the dependent variable with adjustment of potential confounding demographic factors. Zero-order and partial correlation were also calculated from the multivariate regression. Breusch-Pagan test was used to test each regression model for heteroskedasticity of residuals. If heteroskedasticity exists, the regression contains unequal variance and the analysis results may be invalid. All tests were two-tailed, with a significance level of *p* < 0.05. Statistical analysis was done using SPSS Statistic 28.0.

## Results

### Demographics of respondents

We received responses from 1,526 respondents, and 175 did not complete the questionnaires. Eventually, we included 1,332 respondents (completion rate: 87.29%) from 22 provinces, four municipalities and five autonomous regions in China. The five areas with the largest number of respondents were Beijing (*N* = 235, 17.64%), Sichuan Province (*N* = 205, 15.39%), Guangdong Province (*N* = 145, 10.89%), Chongqing (*N* = 105, 7.88%) and Hubei Province (*N* = 73, 5.48%). Table 1 summarizes the demographics of the participants. Sixty-eight percent of participants were 40 years and below. The majority of participants were women (56.8%), had a university education with at least a bachelor degree and above (70.8%), married (58.9%) and living with 3 to 5 people (60.5%). Only 4.1% of respondents were unemployed, and the three most frequent occupations were professional and technical personnel (17.1%), managers of government agencies and public institutions (16.5%) and students (14.6%). The majority of respondents (44%) worked in the office, while 17 and 9.6% worked and studied from home, respectively.

**Table 1:** Demographic data characteristics (*N* = 1,332).

Demographic data	Number (%)
Age	
18–21 years	169 (12.6)
22–30 years	296 (22.2)
31–40 years	442 (33.2)
41–49 years	274 (20.6)
50–59 years	114 (8.6)
Above 60 years	37 (2.8)
Gender	
Male	575 (43.2)
Female	757 (56.8)
Highest education level	
None/Kindergarten	4 (0.3)
Primary school	8 (0.6)
Junior high school	31 (2.3)
Senior high school	88 (6.6)
Junior college	258 (19.4)
University: Bachelor	665 (49.9)
University: Master	232 (17.4)
University: PhD	46 (3.5)
Marital status	
Single or not married	473 (35.5)
Married	785 (58.9)
Divorce or Separated	65 (4.9)
Widowed	9 (0.7)
Household size	
Living alone	139 (10.4)
2 people	331 (24.8)
3 to 5 people	806 (60.5)
6 or more people	56 (4.2)
Do you live with the following family members (Multiple choice)	
Living with a child under 16 years old	540
Living with a child over 16 years old	160
Living with two or more children	191
Living with a healthy older adult	444
Living witan a older adult with poor health	102
Do not live with children or older adult	382
Employment status	
Unemployed or waiting for work	54 (4.1)
Retired	74 (5.6)
Freelancer	137 (10.3)
Managers of government agencies and public institutions	220 (16.5)
Professional and technical personnel	228 (17.1)
Clerical and related personnel	64 (4.8)
Commercial and service personnel	102 (7.7)
Production personnel in agriculture, forestry and fishery	7 (0.5)
Production, transportation and equipment operators	21 (1.6)
Soldier	1 (0.1)
Student	195 (14.6)
Housewife	30 (2.3)
Medical worker, doctor, nurse, examiner, therapist, others	70 (5.3)
Workers related to epidemic prevention, nucleic acid monitoring	3 (0.2)
Self-employed	31 (2.3)
Others	92 (7.1)
Work/study condition	
I am working in the office	587 (44.1)
I am working in a factory	50 (3.8)
I am working from home	226 (17.0)
I do not have a fixed place to work	51 (3.8)
I am working on vacation	46 (3.5)
I am studying from home	128 (9.6)
I am studying in school	23 (1.7)
I am on study leave or vacation	46 (3.5)
Others	175 (13.1)

### Frequencies of responses to health status and questions related to COVID-19 pandemic and dynamic zero-COVID policy

The most common physical symptoms experienced by respondents in the past 14 days were cough (740 respondents), stuffy nose (598 respondents) and coughing up phlegm (594 respondents; see Supplementary Table S2). About 63.3% of respondents related their health status as healthy and relatively healthy. About 72.1% of respondents had at least one episode of COVID-19 infection. About 58.4% were fully vaccinated with no booster shot and one booster shot. The most common items hoarded by respondents were fever medicine (1,041 respondents), cold medicine (980 respondents) and thermometer (866 respondents). About 1% of respondents were admitted to Fangcang hospital due to COVID-19 infection. About 2.9% of respondents had a family or friend who died from COVID-19 infection. About 70.3% of respondents experienced at least one lockdown in their residential areas. About 63.9% of respondents experienced at least 7 days of lockdown in 2022. About 79.5% of respondents were slightly and very worried about their family members getting COVID-19 infection. The measures associated with dynamic zero-COVID policy had the highest percentage of strong agreement of beneficences were home quarantine for people infection with COVID-19 infection (31%), receiving COVID-19 infection and booster shots (25.9%) and close management of small district with infected COVID-19 cases (23.2%; see Supplementary Table S3). About 95.3% of respondents rated mild to very severe impact due to the COVID-19 pandemic and dynamic zero-COVID policy (see Supplementary Table S4).

### Prevalence of COVID-19 over-concern

Table 2 shows the number of participants who scored above the cut-off scores for the Y-BOCS, Illness Anxiety Scale, DASS-21 Scale, IES-R Scale and Insomnia Severity Index. About 27.2% of respondents exhibited significant obsessive-compulsive symptoms, 37.6% demonstrated significant illness anxiety, 20.5% reported significant PTSD symptoms, 27.8% reported significant depressive symptoms, 35.4% reported significant anxiety symptoms, 10.3% reported significant stress symptoms and 7.9% reported clinically significant insomnia. About 54.4% of respondents experienced five or more COVID-19-related symptoms in the past 14 days, and 68.5% of respondents hoarded 5 or more COVID-19-related precautionary items in the past 14 days. Two hundred eighty-three respondents (21.2%) who fulfilled the diagnostic criteria for COVID-19 over-concern by meeting the cut-off for at least 5 of the above symptoms.

**Table 2:** Summary of yale-brown, illness anxiety, DASS-21, IES-R, and insomnia severity index scores among participants (*N* = 1,332).

Psychosocial profile	Mean ± SD [Number (%)]
**Yale-Brown total score**	**11.84 ± 5.71**
Number of participants above YBOCS ^a^ cut-off score	362 (27.2)
**Illness anxiety total score**	**38.81 ± 16.10**
IAS^b^-1: Worry about illness	4.77 ± 2.66
IAS-2: Concerns about pain	5.64 ± 2.82
IAS-3: Health habits	6.42 ± 2.92
IAS-4: Hypochondriacal beliefs	3.44 ± 2.33
IAS-5: Thanatophobia	3.90 ± 3.13
IAS-6: Disease phobia	3.90 ± 3.13
IAS-7: Bodily preoccupation	3.98 ± 2.78
IAS-8: Treatment experience	4.10 ± 2.61
IAS-9: Effects of symptoms	3.73 ± 2.56
Number of participants above IAS cut-off score	501 (37.6)
**IES-R** ^c^ **total score**	**20.30 ± 14.59**
Avoidance Intrusion Hyperarousal	7.38 ± 5.71 7.43 ± 5.59 5.44 ± 4.36
The number of participants above the IES-R cut-off score	273 (20.5)
**DASS-21** ^**e**^ **score**	**23.30 ± 22.91**
Depression Anxiety Stress	7.81 ± 8.10 7.31 ± 7.59 8.19 ± 8.15
Number of participants above the Depression cut-off score	370 (27.8)
Number of participants above the Anxiety cut-off score	471 (35.4)
Number of participants above the Stress cut-off score	137 (10.3)
**Insomnia severity index total score**	**6.49 ± 5.48**
Number of participants above ISI^e^ cut-off score	105 (7.9)
**Participants who experienced five or more COVID-19-related symptoms in the past 14 days**	**725 (54.4)**
**Participants who hoarded five or more COVID-19-related prevention and control items in the past 14 days**	**913 (68.5)**

^a^YBOCS, Yale-Brown Obsessive-compulsive scale. ^b^IAS, Illness Anxiety Scale. ^c^IES-R, Impact of Event Scale-Revised. ^d^DASS-21, Depression, Anxiety, Stress Scale. ^e^ISI, Insomnia Severity Index.

### Regression analysis

Table 3 shows the linear regression analysis using psychological outcomes as dependent variables and demographic data as independent variables. Younger age was significantly associated with higher mean scores in obsessive-compulsive symptoms (*p* < 0.05), post-traumatic stress (*p* < 0.01), depression, anxiety and stress (*p* < 0.001). Male gender (*p* < 0.01) and education (*p* < 0.001) were significantly associated with higher mean scores of illness anxiety. Employment was significantly associated with lower mean scores in obsessive-compulsive symptoms (*p* < 0.05), illness anxiety (*p* < 0.05), depression, anxiety and stress (*p* < 0.05) and insomnia (*p* < 0.05). Marital status and household size were not associated with psychological outcomes (*p* > 0.05). Breusch-Pagan test showed the presence of heteroskedasticity in the regression model using DASS-21 as dependent variable (*p* < 0.05; see Supplementary Table S5).

**Table 3:** The linear regression analysis using psychological outcomes as dependent variables and demographic data as independent variables (*N* = 1,332).

Demographic data	YBOCS^a^	IAS^b^	IES-R^c^	DASS-21^d^	ISI^e^	Collinearity
	B^f^ (SE)	B (SE)	B (SE)	B (SE)	B (SE)	VIF^g^
Age	−0.043 (0.018)*	−0.083 (0.051)	−0.127 (0.047)**	−0.347 (0.073)***	0.000 (0.018)	1.729
Gender (Female)	0.295 (0.316)	−2.429 (0.886)**	−0.034 (0.809)	0.855 (1.259)	0.176 (0.304)	1.007
Education level	0.269 (0.154)	1.663 (0.431)***	0.135 (0.393)	0.405 (0.612)	−0.028 (0.148)	1.080
Marital status	0.586 (0.339)	1.322 (0.951)	1.107 (0.868)	1.760 (1.352)	−0.162 (0.326)	1.632
Household size	0.334 (0.216)	0.717 (0.606)	0.518 (0.553)	1.330 (0.862)	0.154 (0.208)	1.029
Employment status	−0.780 (0.371)*	−1.516 (1.040)	−1.530 (0.950)	−3.569 (1.479)*	−0.872 (0.357)*	1.127

^a^YBOCS, Yale-Brown Obsessive-compulsive scale. ^b^IAS, Illness Anxiety Scale. ^c^IES-R, Impact of Event Scale-Revised. ^d^DASS-21, Depression, Anxiety, Stress Scale. ^e^ISI, Insomnia Severity Index. ^f^B, regression coefficient; SE, standard error. ^g^VIF, variance inflation factor. *p < 0.05. **p < 0.01. ***p < 0.001.

Table 4 shows the linear regression analysis using psychological outcomes as dependent variables and physical health status as independent variables. The presence of 5 or more physical symptoms was significantly associated with higher mean scores in illness anxiety (*p* < 0.001), post-traumatic stress (*p* < 0.01), depression, anxiety and stress (*p* < 0.001) and insomnia (*p* < 0.001). Poor self-health status was significantly associated with higher mean scores in obsessive-compulsive symptoms (*p* < 0.001), illness anxiety (*p* < 0.001), post-traumatic stress symptoms (*p* < 0.001), DASS-21 (*p* < 0.001) and insomnia (*p* < 0.001). Hoarding of 5 or more COVID-19-related precautionary items was associated with higher mean scores in post-traumatic stress symptoms (*p* < 0.001), depression, anxiety and stress (*p* < 0.001). The higher number of days under lockdown was significantly associated with higher mean scores for obsessive-compulsive symptoms (*p* < 0.05), illness anxiety (*p* < 0.05), post-traumatic stress (*p* < 0.001), depression, anxiety and stress (*p* < 0.05) and insomnia (*p* < 0.05). More concern about family members getting COVID-19 infection was significantly associated with higher mean scores in obsessive-compulsive symptoms (*p* < 0.001), illness anxiety (*p* < 0.001), post-traumatic stress symptoms (*p* < 0.001), depression, anxiety and stress (*p* < 0.001) and insomnia (*p* < 0.001). The number of past COVID-19 infections, COVID-19 vaccination status, previous admission to Fangcang Hospitaland death of a family member or friend due to COVID-19 were not associated with any psychological outcome (*p* > 0.05). Breusch-Pagan test showed absence of heteroskedasticity for all regression models below (*p* > 0.05; see Supplementary Table S5).

**Table 4:** The linear regression analysis using psychological outcomes as dependent variables and physical health factors as independent variables (*N* = 1,332).

Physical health status	YBOCS^a^	IAS^b^	IES-R^c^	DASS-21^d^	ISI^e^	Collinearity
	B^f^ (SE)	B (SE)	B (SE)	B (SE)	B (SE)	VIF^g^
5 or more physical symptoms	0.494 (0.308)	4.263 (0.813)***	2.063 (0.772)**	6.561 (1.221)***	1.066 (0.296)***	1.050
Self-rated health status	0.954 (0.236)***	4.242 (0.622)***	2.439 (0.591)***	3.558 (0.934)***	1.174 (0.226)***	1.013
Number of past COVID-19 infections	−0.083 (0.104)	−0.401 (0.274)	−0.289 (0.260)	−0.432 (0.412)	−0.174 (0.100)	1.017
COVID-19 vaccination status	0.206 (0.141)	0.205 (0.372)	−0.213 (0.353)	−0.662 (0.558)	−0.124 (0.135)	1.003
5 or more items hoarded	−0.083 (0.328)	1.168 (0.867)	−2.809 (0.824)***	−5.113 (1.302)***	−0.361 (0.316)	1.038
Admission to Fangcang Hospital	0.135 (1.538)	3.258 (4.061)	2.891 (3.857)	3.095 (6.100)	1.401 (1.479)	1.021
Family or friend died from COVID-19	1.405 (0.913)	1.529 (2.410)	3.580 (2.289)	6.718 (3.620)	1.190 (0.878)	1.031
Number to times residential area been locked down	0.409 (0.123)***	0.518 (0.326)	0.454 (0.309)	1.074 (0.489)*	0.236 (0.119)*	1.403
Number of days being lockdown in 2022	0.307 (0.131)*	0.762 (0.345)*	1.111 (0.328)***	1.033 (0.518)*	0.251 (0.126)*	1.399
Concerned about family members getting COVID-19	1.298 (0.174)***	6.460 (0.460)***	4.488 (0.437)***	5.706 (0.691)***	1.129 (0.168)***	1.029

^a^YBOCS, Yale-Brown Obsessive-compulsive scale. ^b^IAS, Illness Anxiety Scale. ^c^IES-R, Impact of Event Scale-Revised. ^d^DASS-21, Depression, Anxiety, Stress Scale. ^e^ISI, Insomnia Severity Index. ^f^B, regression coefficient; SE, standard error. ^g^VIF, variance inflation factor. *p < 0.05. **p < 0.01. ***p < 0.001.

Table 5 shows the linear regression analysis using psychological outcomes as dependent variables and views toward dynamic zero-COVID policy as independent variables. Respondents who viewed COVID vaccination as beneficial were significantly associated with higher mean scores in obsessive-compulsive symptoms (*p* < 0.01), illness anxiety (*p* < 0.01), post-traumatic stress (*p* < 0.001), depression, anxiety and stress (*p* < 0.001) and insomnia (*p* < 0.001). In contrast, respondents who viewed working from home as beneficial were significantly associated with lower illness anxiety (*p* < 0.01) but higher insomnia (*p* < 0.05). Respondents who viewed the limitation on sales of antipyretics and other medications as non-beneficial were significantly associated with a higher level of illness anxiety (*p* < 0.01) and insomnia (*p* < 0.05). Respondents who viewed limiting people in public dining areas as beneficial were significantly associated with lower mean scores in obsessive-compulsive symptoms (*p* < 0.05), illness anxiety (*p* < 0.001) and post-traumatic stress (*p* < 0.01). Respondents who viewed wearing face mask all the time as beneficial was significantly associated with a lower mean score in insomnia (*p* < 0.01). Views toward massive nucleic acid testing, home quarantine, close management of the small district, electronic health pass, travel restriction, avoidance of face-to-face contact and closure of indoor leisure facilities were not associated with any psychological outcome (*p* > 0.05). Breusch-Pagan test showed the presence of heteroskedasticity in the regression models using DASS-21 and ISI as dependent variables (*p* < 0.05; see Supplementary Table S5).

**Table 5:** The linear regression analysis using psychological outcomes as dependent variables and views toward the dynamic zero-COVID policy as independent variables (*N* = 1,332).

Opinions	YBOCS^a^	IAS^b^	IES-R^c^	DASS-21^d^	ISI^e^	Collinearity
	B^f^ (SE)	B (SE)	B (SE)	B (SE)	B (SE)	VIF^g^
Massive nucleic acid testing is beneficial	−0.261 (0.227)	−1.094 (0.630)	−0.082 (0.578)	−0.223 (0.915)	0.016 (0.216)	2.263
Home quarantine for people with COVID-19 is beneficial	0.053 (0.224)	0.280 (0.621)	−0.033 (0.570)	0.047 (0.902)	0.182 (0.213)	1.478
Home quarantine for people with close contact but no COVID-19 is beneficial	0.244 (0.185)	0.567 (0.512)	−0.149 (0.470)	−0.127 (0.744)	0.259 (0.176)	1.685
Close management of a small district with COVID-19 cases is beneficial	0.302 (0.250)	−0.953 (0.693)	−1.054 (0.636)	−0.760 (1.006)	−0.069 (0.238)	2.384
Receiving COVID-19 vaccination and booster shots is beneficial	0.647 (0.197)**	1.416 (0.546)**	2.013 (0.501)***	2.859 (0.793)***	0.882 (0.187)***	1.350
The use of electronic health pass is beneficial	0.243 (0.255)	−0.215 (0.709)	0.461 (0.650)	0.736 (1.030)	−0.321 (0.243)	2.862
Travel restriction is beneficial	0.004 (0.211)	0.665 (0.584)	−0.325 (0.536)	0.015 (0.849)	−0.294 (0.200)	2.307
Attending online classes and avoid face-to-face classes is beneficial	0.167 (0.194)	0.623 (0.540)	0.553 (0.495)	−0.410 (0.784)	−0.188 (0.185)	1.902
Working from home is beneficial	0.013 (0.215)	−1.650 (0.598)**	0.047 (0.548)	0.116 (0.868)	0.479 (0.205)*	1.725
Limitation on the sale of antipyretics and medication to treat influenzas over the counter is beneficial	0.281 (0.156)	1.211 (0.433)**	0.052 (0.397)	−0.990 (0.629)	0.327 (0.148)*	1.364
Closure of indoor public spaces such as bars, KTV, and swimming pools is beneficial	−0.134 (0.234)	−0.111 (0.650)	−0.476 (0.597)	−0.180 (0.945)	−0.161 (0.223)	2.520
Limiting people in dining areas and encouraging customers to take away is beneficial	−0.590 (0.260)*	−2.514 (0.723)***	−1.767 (0.663)**	−1.049 (1.050)	−0.153 (0.248)	2.481
Wearing face mask all the time is beneficial	−0.354 (0.245)	−0.912 (0.680)	−0.347 (0.624)	−1.159 (0.988)	−0.644 (0.233)**	2.210

^a^YBOCS, Yale-Brown Obsessive-compulsive scale. ^b^IAS, Illness Anxiety Scale. ^c^IES-R, Impact of Event Scale-Revised. ^d^DASS-21, Depression, Anxiety, Stress Scale. ^e^ISI, Insomnia Severity Index. ^f^B, regression coefficient; SE, standard error. ^g^VIF, variance inflation factor. *p < 0.05. **p < 0.01. ***p < 0.001.

Table 6 shows the linear regression analysis using psychological outcomes as dependent variables and the perceived impact of measures associated with dynamic zero-COVID policy as independent variables. The greater perceived impact on lives was significantly associated with higher mean scores in obsessive-compulsive symptoms (*p* < 0.001), illness anxiety (*p* < 0.001), post-traumatic stress symptoms (*p* < 0.001), DASS-21 (*p* < 0.01) and insomnia (*p* < 0.001). The greater perceived impact on income, employment and studies was significantly associated with obsessive-compulsive symptoms (*p* < 0.001), post-traumatic symptoms (*p* < 0.01), depression, anxiety and stress (*p* < 0.001). The perceived impact on the family was significantly associated with higher mean scores in obsessive-compulsive symptoms (*p* < 0.001), illness anxiety (*p* < 0.001), post-traumatic stress symptoms (*p* < 0.01), depression, anxiety and stress (*p* < 0.01) and insomnia (*p* < 0.05). The perceived impact of traveling was significantly associated with obsessive-compulsive symptoms (*p* < 0.001) and illness anxiety (*p* < 0.05). Breusch-Pagan test showed the presence of heteroskedasticity for all regression models below (*p* < 0.05; see Supplementary Table S5).

**Table 6:** The linear regression analysis using psychological outcomes as dependent variables and the perceived impact of measures associated with dynamic zero-COVID policy as independent variables (*N* = 1,332).

Perceived impact of measures	YBOCS^a^	IAS^b^	IES-R^c^	DASS-21^d^	ISI^e^	Collinearity
	B^f^(SE)	B (SE)	B (SE)	B (SE)	B (SE)	VIF^g^
Impact on lives	1.398 (0.197)***	3.184 (0.601)***	2.003 (0.554)***	2.514 (0.872)**	0.946 (0.209)***	1.861
Impact on incomes, employment, and studies	0.696 (0.159)***	0.282 (0.484)	1.459 (0.446)**	2.579 (0.703)***	0.234 (0.169)	1.667
Impact on families	0.632 (0.154)***	1.571 (0.471)***	1.329 (0.433)**	2.125 (0.683)**	0.398 (0.164)*	1.629
Impact on leisure and business trips	0.507 (0.149)***	0.980 (0.454)*	−0.135 (0.418)	−0.344 (0.659)	−0.055 (0.158)	1.637
The latest health information related to COVID-19 are clear and correct	0.315 (0.150)*	0.961 (0.457)*	0.563 (0.421)	1.068 (0.663)	0.428 (0.159)**	1.006

^a^YBOCS, Yale-Brown Obsessive-compulsive scale. ^b^IAS, Illness Anxiety Scale. ^c^IES-R, Impact of Event Scale-Revised. ^d^DASS-21, Depression, Anxiety, Stress Scale. ^e^ISI, Insomnia Severity Index. ^f^B, regression coefficient; SE, standard error. ^g^VIF, variance inflation factor. *p < 0.05. **p < 0.01. ***p < 0.001.

Table 7 shows the linear regression analysis between COVID-19 over-concern and significant independent variables identified by previous regression analysis after adjustment for age, gender, education level and employment status. Poor self-rated health status (*p* < 0.001), number of days under lockdown in 2022 (*p* < 0.01), concerns about family members contracting COVID-19 infection (*p* < 0.001), perceived usefulness of receiving COVID-19 vaccine and booster shot (*p* = 0.011) were significantly associated with the development of COVID-19 over-concern. In contrast, views on working from home, views on the limitation on sales of antipyretics and medication, and views on limitations on public dining were not associated with the developing of COVID-19 over-concern (*p* > 0.05). Breusch-Pagan test showed absence of heteroskedasticity in the regression model (*p* > 0.05).

**Table 7:** Linear regression analysis between COVID-19 over-concern and independent variables (*N* = 1,332) after adjustment of confounding demographic factors.

Independent variables	COVID-19 over-concern^a^				
	B^a^(SE)	Zero-order correlation	Partial correlation	*p*-value	VIF^b^
Poor self-rated health status	0.077 (0.017)	0.129	0.123	<0.001***	1.004
Number of days under lockdown in 2022	0.025 (0.008)	0.098	0.083	0.002**	1.007
Concerned about family members getting COVID-19	0.081 (0.013)	0.178	0.167	<0.001***	1.088
Receiving COVID-19 vaccination and booster shots is beneficial	0.033 (0.013)	0.037	0.070	0.011*	1.200
Working from home is not beneficial	0.006 (0.013)	0.040	0.012	0.661	1.246
Limitation on the sale of antipyretics and medication to treat influenzas over the counter is not beneficial	0.004 (0.010)	0.012	0.012	0.672	1.194
Limiting people in public dining areas and encouraging customers to take away is not beneficial	0.009 (0.014)	0.054	0.019	0.498	1.405

Adjusted with confounding factors: Age, Gender, Education level, Employment status. ^a^B, regression coefficient; SE, standard error. ^b^VIF, variance inflation factor. *p < 0.05, **p < 0.01, ***p < 0.001.

## Discussion

This study was conducted to explore COVID-19 over-concern following the removal of stringent restrictive measures in a country implementing the dynamic zero-COVID policy. Data collection occurred during a critical window from December 25 to 27, 2022, which followed closely after the peak of COVID-19 infections in China on 22 December 2022, when the Chinese Center for Disease Control and Prevention reported a peak of 6.94 million positive cases, which subsequently began to decline (35). COVID-19 over-concern could be considered a new type of hypochondriasis during this period (36). Our study featured 1,332 respondents from 31 regions in China, boasting a robust 87.29% response rate. This diverse sample, comprising individuals of different ages, genders, educational backgrounds, marital statuses, and living arrangements, contributes to the depth and breadth of our research findings. According to this study, after the cancelation of the measures adopted by the dynamic zero-COVID policy in China, there were 283 respondents (21.2%) who fulfilled at least five of the diagnostic criteria for COVID-19 over-concern. This finding supports hypotheses 1 (The COVID-19 over-concern was a common condition and the prevalence would be higher than 10% when the dynamic zero-COVID measures were lifted). This study identified that poor self-rated health status, concerns about family members contracting COVID-19 infection, perceived usefulness of receiving COVID-19 vaccine and booster shot, negative impact on incomes, employment and studies and negative impact on families were significantly associated with the development of COVID-19 over-concern after adjustment of confounding demographic factors and these associations warranted further discussion.

Young age was associated with the development of COVID-19 over-concern after the cancelation of measures associated with the dynamic zero-COVID policy. In this study, young age was specifically associated with more severe obsessive-compulsive symptoms and post-traumatic stress, after the cancelation of measures associated with the dynamic zero- COVID policy. This finding was not surprising as the academic and social lives of young Chinese were more affected by the COVID-19 pandemic due to the prolonged closure of school and cancelation of extra-curricular activies (37), long periods of online learning (38, 39), disruption of public examinations (11), suspension of cross border education between territories (40), not being able to return to hometown or village during holidays (37), poor social support from peers (39), and social isolation due to quarantine of self, friends and family members (39, 41). Because of the above school arrangement and lifestyle changes, young Chinese were deprived of the opportunities to develop social and interpersonal skills during the pandemic. Consequently, they might experience the greatest psychological impact following the cancelation of measures associated with the dynamic zero-COVID policy, as they foresaw challenges in readjusting to their previous lifestyles with more face-to-face contact and concerns about the potential risk of contracting COVID-19 without restrictive measures.

The most common physical symptoms experienced by respondents in the past 14 days were cough, stuffy nose and coughing up phlegm. It is not surprising that poor self-rated health status and concerns about family members contracting COVID-19 infection were associated with the development of COVID-19 over-concern after the cancelation of measures associated with the dynamic zero COVID policy. This finding supported hypothesis 2 (Higher severity of physical symptoms would be associated with higher severity of psychological symptoms, including obsessive-compulsive symptoms, illness anxiety, depression, anxiety, stress, PTSD symptoms and insomnia). This study found that poor self-rated health status and more concern about family members getting COVID-19 were significantly associated with higher mean scores in obsessive-compulsive symptoms, illness anxiety, post-traumatic stress symptoms, depression, anxiety and stress and insomnia. While most of the research focuses on mental health status, there is a paucity of research data on the Chinese population about the impact of poor self-rated physical status and concerns about family members during the COVID-19 pandemic. A previous global research found that poor self-rated physical health status is a mediator between the COVID-10 pandemic and adverse mental health status (10). After the cancelation of the dynamic zero-COVID measures, people with poor self-rated health status and concerns about family members faced challenges in two folds: (1) an increased risk of contracting COVID-19 infection; (2) a reduction in healthcare system resources available to manage non-COVID-19 diseases due to the strain imposed by an overload of COVID-19 cases.

It was unexpected that the perceived usefulness of receiving the COVID-19 vaccine and booster shot was significantly associated with the development of COVID-19 over-concern after the cancelation of measures associated with the dynamic zero-COVID policy. This finding suggests that people might perceive the COVID-19 vaccine and booster as useful but still lack confidence that the vaccine could prevent future strains of COVID-19 infection. Global acceptance of COVID-19 vaccines depends on several factors related to psychology, society, and the vaccines themselves. A previous study found that low confidence in the COVID-19 vaccine might negatively influence people’s health behaviors during the COVID-19 pandemic (42). Since China did not have an mRNA vaccine available at the time of the study, participants may have had unmet expectations regarding the vaccine’s effectiveness (43), particularly against future strains and mutations of coronavirus. The confidence might improve as the Chinese-made mRNA vaccine against Omicron strains started production in 2023 (44).

Finally, negative impacts on incomes, employment, studies and families were significantly associated with COVID-19 over-concern after the cancelation of measures associated with the dynamic zero-COVID policy. This finding partially supports hypothesis 3 (The severity of COVID-19 over-concern among individuals was positively associated with how much they perceive various aspects of their lives being affected). In this study, most respondents rated mild to very severe impact due to the COVID-19 pandemic and dynamic zero-COVID policy. Unlike citizens in other countries who sought to lift COVID-19 restrictions to facilitate the return of their incomes, jobs, educational pursuits, and family life to pre-pandemic standards, our findings suggest that the Chinese individuals were worried about the further negative impact on the economy, work, study and family if the COVID-19 outbreak was out of control and further prolonged their suffering. These findings align with the outcomes of a previous multinational study, highlighting differences between Chinese citizens and their counterparts from other countries in this regard (45).

After the dynamic zero-COVID policy was lifted, there was a significant increase in COVID-19 cases (896%, from 21.54 to 49.01 per million people) and deaths (127%, an absolute change of 27.46 per million people) in the following 6 weeks (46, 47). The notable rise in infections and deaths coincided with the public’s overconcern, suggesting that the COVID-19 over-concern was not just a psychopathological phenomenon but also a realistic reaction to the rapidly changing situation.

### Implications for policy changes

The findings of this study have the following implications for policy changes and preparator work for resuming pre-pandemic life. First, around one-fifth of the population developed COVID-19 over-concern when there was a sudden change in the dynamic zero-COVID policy. Health authorities should form a task force to roll out targeted online interventions, like cognitive behavioral therapy (48), for COVID-19 over-concern, along with monitoring their effectiveness. Second, the Ministry of education, schools and local psychological services should offer group social and interpersonal skill training for young Chinese. These programs should be tailored to help young people adapt to increased face-to-face interactions, reflecting the shift from virtual to in-person communication in the post-pandemic era. Third, a rapid response from the public health system targets people with poor health status. This includes ensuring equitable access to medical services for both COVID-19-related and unrelated health issues, with an emphasis on vulnerable populations (49). Fourth, health authorities could adopt the enhancing Confidence in vaccines, reducing Complacency to health risks of COVID-19 infection, enhancing Convenience to receiving vaccines and accurate COVID-19 Communications (4C) model to enhance confidence in future COVID-19 vaccines (43). A clear communication strategy and community involvement are key to building trust in these programs. Fifth, the government should reduce the impact on income, employment, studies and families in future pandemics. Policies should be developed to provide support and resources to minimize lifestyle disruptions, including policies for financial and mental health support and flexible work and education arrangements. Additionally, it would be crucial to conduct these psychological interventions ethically, respecting individual rights and privacy and adhering to ethical standards.

### Limitations of this study

This study has several limitations. First, adopting the snowball sampling strategy due to the urgency following the cancelation of the dynamic zero-COVID policy introduced a key limitation of potential selection bias, as respondent recruitment was not random. To mitigate this, we initiated recruitment from multiple, diverse recruitment sites, but the possibility of bias still persists. Consequently, the study population might not fully represent the broader population, including those without digital access. Additionally, reliance on participant honesty for exclusion criteria, without the ability to verify their medical and psychiatric history, further limit the findings of this study.

Second, the study identifies associations but does not establish causality. The cross-sectional nature of this study limits our ability to establish causality between the observed factors and COVID-19 over-concern. These findings are useful for hypothesis formation, but longitudinal studies are needed to establish causality. The sudden cancelation of the zero-COVID policy restricted our ability to gather pre-cancelation data or conduct a long-term comparison. Also, due to the anonymity of respondents, we could not collect personal details for future follow-up studies.

Third, this study used self-reports to assess psychological symptoms like anxiety, depression, stress, and COVID-19 over-concern. Self-reported data can be less reliable than clinical assessments, as participants might give biased answers or lack self-awareness regarding their mental health. Future research should include clinical evaluations to collect more accurate data.

Fourth, despite COVID-19 overconcern being a new research area without established criteria, we rigorously evaluated our proposed criteria. We assessed the Cronbach’s alpha reliability, Split-half reliability, CVI, and Kappa values for our survey, yielding high scores that indicate strong reliability and validity. These results suggest the robustness of our criteria, though future research might refine them further. Despite the above limitations, our study offers unique insights into COVID-19 over-concern prevalence and its associated factors, contributing significantly to global data during the pandemic.

## Conclusion

After canceling measures associated with the dynamic zero-COVID policy in China, around 21.2% of respondents fulfilled the diagnostic criteria for COVID-19 over-concern. This study identified that poor self-rated health status, concerns about family members contracting COVID-19 infection, perceived usefulness of receiving COVID-19 vaccine and booster shot, negative impact on incomes, employment and studies and negative impact on families were significantly associated with the development of COVID-19 over-concern after adjustment of confounding demographic factors. Our findings will help health authorities formulate future policies to prepare the public to cope with the transition between different phases during a pandemic.

## Data availability statement

The raw data supporting the conclusions of this article will be made available by the authors, without undue reservation.

## Ethics statement

The studies involving humans were approved by Institutional Review Board (IRB) of the Huaibei Normal University, China (HBS-FDX-2022-012). The studies were conducted in accordance with the local legislation and institutional requirements. The participants provided their written informed consent to participate in this study.

## Author contributions

FH: Conceptualization, Data curation, Investigation, Methodology, Resources, Writing – original draft, Writing – review & editing. ZZ: Conceptualization, Methodology, Writing – review & editing. SL: Conceptualization, Methodology, Writing – review & editing. S-KC: Data curation, Formal analysis, Writing – original draft. DZ: Data curation, Writing – review & editing. WT: Data curation, Writing – review & editing. XT: Funding–acquisition, Project administration, Supervision, Writing – review & editing. RH: Conceptualization, Methodology, Writing – original draft, Writing – review & editing.
