# Supplementary material for: The emergence of COVID-19 over-concern immediately after the cancelation of the measures adopted by the dynamic zero-COVID policy in China
Source: Front Public Health. 2024 Jan 5;11:1319906. doi: 10.3389/fpubh.2023.1319906 (PMC10796473; doi:10.3389/fpubh.2023.1319906)
Supplement: Supplementary file 1 [file Table_1.DOCX]

**S1 Table. Internal Consistency, Split-Half Reliability and Content Validity for the questionnaire**

| Part | Cronbach’s alpha eliability | Split-half reliability | Content Validity Index | Kappa value |
| --- | --- | --- | --- | --- |
| Physical symptoms in the past 14 days | 0.893 | 0.725 | 0.912 | 0.905 |
| Stockpiling supplies for potential COVID-19 infection risk | 0.839 | 0.782 | 0.958 | 0.953 |
| Yale-Brown Obsessive Compulsive scale with specific reference to measures related to the dynamic zero-COVID policy | 0.833 | 0.821 | 0.857 | 0.849 |
| the illness attitude scale with specific reference to COVID-19 infection (IAS) | 0.911 | 0.827 | 1.000 | 1.000 |
| Impact of Event Scale–Revised (IES-R) | 0.955 | 0.933 | 1.000 | 1.000 |
| Depression, Anxiety and Stress scale -21 items (DASS-21)- DEPRESSION | 0.901 | 0.835 | 1.000 | 1.000 |
| Depression, Anxiety and Stress scale -21 items (DASS-21) ANXIETY | 0.894 | 0.858 | 1.000 | 1.000 |
| Depression, Anxiety and Stress scale -21 items (DASS-21) STRESS | 0.900 | 0.882 | 1.000 | 1.000 |
| Insomnia Severity Index (ISI) | 0.921 | 0.881 | 1.000 | 1.000 |
| Total | 0.967 | 0.783 | 0.967 | 0.967 |
