# Supplementary material for: The emergence of COVID-19 over-concern immediately after the cancelation of the measures adopted by the dynamic zero-COVID policy in China
Source: Front Public Health. 2024 Jan 5;11:1319906. doi: 10.3389/fpubh.2023.1319906 (PMC10796473; doi:10.3389/fpubh.2023.1319906)
Supplement: Supplementary file 2 [file Table_2.DOCX]

**S2 Table. Responses to physical health status in the past 14 days, information related to COVID-19 infection and vaccination situations (N = 1332)**

| **Physical Health status** | **Number (%)** |
| --- | --- |
| **Symptoms in the past 14 days (A respondent can report more than 1 symptom)** |  |
| Heat all over body  Fatigue  Sore limbs  Chills  Loss of appetite  Fever < 38.5°C  Fever ≥ 38.5°C  Cough  Sore throat  Stuffy nose  Runny nose  Coughing up phlegm  Chest tightness  Out of breath  Shortness of breath  Breathing difficulty  Vomiting  Nausea  Diarrhea  Dizziness  Headache  Drowsiness  Lethargy  Coma  Loss of smell  Loss of taste  None of the above symptoms | 524  564  531  403  339  273  427  740  526  598  488  594  189  112  149  83  89  142  128  278  324  265  97  14  213  205  271 |
| **Self-rated health status in the past 14 days** |  |
| Healthy or relatively healthy  Average health condition  Poor or very poor health  Have chronic disease | 843 (63.3)  426 (32.0)  40 (3.0)  23 (1.7) |
| **Number of past COVID-19 infections** |  |
| Never  Once  Twice  Three times  Four times or more  Don’t know or not sure | 372 (27.9)  793 (59.5)  11 (0.8)  0 (0)  2 (0.2)  154 (11.6) |
| **COVID-19 vaccination status** |  |
| Not vaccinated against COVID-19  Received at least 1 injection, but failed to complete the vaccination as required  Fully vaccinated, but no booster shot  Fully vaccinated with 1 booster shot  Fully vaccinated with 2 booster shots | 109 (8.2)  72 (5.4)  373 (28.0)  588 (44.1)  190 (14.3) |
| **Hoarding of items related to COVID-19 infection (A respondent can hoard more than 1 item)** |  |
| Fever medicine  Painkillers  Cold medicine  Lian Hua Qing Wen and other Chinese patent medicines for prevention and treatment of COVID-19  Chinese herbal decoction  Antibiotics (anti-inflammatory drugs)  Antiviral drugs  Vitamins  Thermometer  Alcohol disinfectant spray, alcohol hand sanitizer gel  Disinfectant  UV lamp  Antibody detection kit  N95 mask  Surgical mask  Food  Other necessities  None of the above items | 1041  655  980  618  142  340  183  394  866  749  482  71  392  814  754  613  291  37 |
| **Situations and concerns related to COVID-19** |  |
| **I was hospitalized or sent to a Fangcang hospital due to COVID-19 infection** |  |
| Yes | 13 (1.0) |
| **A family or friend died from COVID-19 infection** |  |
| Yes | 38 (2.9) |
| **How many times has your residential area been locked down?** |  |
| None  Once  Twice  3 times  4 times or more | 396 (29.7)  300 (22.5)  277 (20.8)  127 (9.5)  232 (17.4) |
| **How many days have you been locked down at home in 2022?** |  |
| <7 days  7 – 14 days  14 – 28 days  1 month – 2 months  2 months or more | 481 (36.1)  295 (22.1)  228 (17.1)  201 (15.1)  127 (9.5) |
| **Concerned about family members getting COVID-19** |  |
| No family members  Not worried at all  Not very worried  Slightly worried  Very worried | 10 (0.8)  53 (4.0)  210 (15.8)  537 (40.3)  522 (39.2) |
