# Supplementary material for: The emergence of COVID-19 over-concern immediately after the cancelation of the measures adopted by the dynamic zero-COVID policy in China
Source: Front Public Health. 2024 Jan 5;11:1319906. doi: 10.3389/fpubh.2023.1319906 (PMC10796473; doi:10.3389/fpubh.2023.1319906)
Supplement: Supplementary file 3 [file Table_3.DOCX]

**S3 Table. Views towards measures associated with dynamic zero-COVID policy among respondents (N = 1332)**

| **Opinions** | **Number (%)** |
| --- | --- |
| **Massive routine nucleic acid testing is beneficial** |  |
| Strongly agree  Agree  Neutral  Disagree  Strongly disagree | 280 (21.0)  482 (36.2)  415 (31.2)  99 (7.4)  56 (4.2) |
| **Home quarantine for people infected with COVID-19 infection is beneficial** |  |
| Strongly agree  Agree  Neutral  Disagree  Strongly disagree | 413 (31.0)  647 (48.6)  217 (16.3)  35 (2.6)  20 (1.5) |
| **Home quarantine for people with close contact but not infected with COVID-19 is beneficial** | |
| Strongly agree  Agree  Neutral  Disagree  Strongly disagree | 206 (15.5)  397 (29.8)  425 (31.9)  236 (17.7)  68 (5.1) |
| **Close management of a small district with infected COVID-19 cases is beneficial** | |
| Strongly agree  Agree  Neutral  Disagree  Strongly disagree | 309 (23.2)  571 (42.9)  326 (24.5)  95 (7.1)  31 (2.3) |
| **Receiving COVID-19 vaccination and booster shots are beneficial** |  |
| Strongly agree  Agree  Neutral  Disagree  Strongly disagree | 345 (25.9)  577 (43.3)  333 (25.0)  46 (3.5)  31 (2.3) |
| **The use of electronic health pass is beneficial** |  |
| Strongly agree  Agree  Neutral  Disagree  Strongly disagree | 251 (18.8)  529 (39.7)  374 (28.1)  121 (9.1)  57 (4.3) |
| **Restricting travel is beneficial** |  |
| Strongly agree  Agree  Neutral  Disagree  Strongly disagree | 171 (12.8)  346 (26.0)  453 (34.0)  258 (19.4)  104 (7.8) |
| **Attending online classes and avoiding face-to-face classes is beneficial** | |
| Strongly agree  Agree  Neutral  Disagree  Strongly disagree | 163 (12.2)  389 (29.2)  444 (33.3)  238 (17.9)  98 (7.4) |
| **Working from home is beneficial** |  |
| Strongly agree  Agree  Neutral  Disagree  Strongly disagree | 271 (20.3)  558 (41.9)  374 (28.1)  100 (7.5)  29 (2.2) |
| **Limitation on the sales of antipyretics and medication to treat influenza over the counter is beneficial** | |
| Strongly agree  Agree  Neutral  Disagree  Strongly disagree | 112 (8.4)  285 (21.4)  381 (28.6)  371 (27.9)  183 (13.7) |
| **Closure of indoor public spaces such as bars, KTV, and swimming pools is beneficial** | |
| Strongly agree  Agree  Neutral  Disagree  Strongly disagree | 240 (18.0)  516 (38.7)  368 (27.6)  147 (11.0)  61 (4.6) |
| **Limiting people in dining areas and encouraging customers to take away is beneficial** | |
| Strongly agree  Agree  Neutral  Disagree  Strongly disagree | 246 (18.5)  667 (50.1)  285 (21.4)  95 (7.1)  39 (2.9) |
| **Wearing face mask all the time is beneficial** | |
| Strongly agree  Agree  Neutral | 303 (22.7)  631 (47.4)  284 (21.3) |
| Disagree  Strongly disagree | 78 (5.9)  36 (2.7) |
