# Supplementary material for: The emergence of COVID-19 over-concern immediately after the cancelation of the measures adopted by the dynamic zero-COVID policy in China
Source: Front Public Health. 2024 Jan 5;11:1319906. doi: 10.3389/fpubh.2023.1319906 (PMC10796473; doi:10.3389/fpubh.2023.1319906)
Supplement: Supplementary file 4 [file Table_4.DOCX]

**S4 Table. The impact of the COVID-19 pandemic and dynamic zero- COVID policy measures on respondents (N = 1332)**

| **COVID-19 impact** | **Number (%)** |
| --- | --- |
| **The COVID-19 pandemic and dynamic zero-COVID policy has affected my life** | |
| None  Mild  Moderate  Severe  Very severe | 62 (4.7)  317 (23.8)  574 (43.1)  296 (22.2)  83 (6.2) |
| **The COVID-19 pandemic and dynamic zero-COVID policy has affected my income, employment, or studies** | |
| None  Mild  Moderate  Severe  Very severe | 177 (13.3)  374 (28.1)  441 (33.1)  244 (18.3)  96 (7.2) |
| **The COVID-19 pandemic and dynamic zero-COVID policy has affected my family** | |
| None  Mild  Moderate  Severe  Very severe | 154 (11.6)  380 (28.5)  442 (33.2)  228 (17.1)  128 (9.6) |
| **The COVID-19 pandemic and dynamic zero-COVID policy has affected my travel and business trips** | |
| None  Mild  Moderate  Severe  Very severe | 133 (10.0)  271 (20.3)  397 (29.8)  356 (26.7)  175 (13.1) |
| **The latest health information related to COVID-19 pandemic are clear and correct** | |
| Strongly disagree  Disagree  Neutral  Agree  Strongly agree | 135 (10.1)  254 (19.1)  662 (49.7)  255 (19.1)  26 (2.0) |
