# Supplementary material for: The emergence of COVID-19 over-concern immediately after the cancelation of the measures adopted by the dynamic zero-COVID policy in China
Source: Front Public Health. 2024 Jan 5;11:1319906. doi: 10.3389/fpubh.2023.1319906 (PMC10796473; doi:10.3389/fpubh.2023.1319906)
Supplement: Supplementary file 5 [file Table_5.DOCX]

**S5 Table. Breusch-Pagan test for Heteroskedasticity of residuals of linear regression models**

| **Dependent variables** | **YBOCS^a^** | **IAS^b^** | **IES-R^c^** | **DASS-21^d^** | **ISI^e^** |
| --- | --- | --- | --- | --- | --- |
|  | **P-Value** | | | | |
| **Independent variables** |  |  |  |  |  |
| **Demographics** | 0.365 | 0.214 | 0.181 | 0.020* | 0.051 |
| **Physical health** | 0.407 | 0.326 | 0.111 | 0.059 | 0.077 |
| **Views towards policy** | 0.926 | 0.226 | 0.052 | 0.007* | 0.006* |
| **Perceived impact of measures** | <0.001* | 0.002* | <0.001* | <0.001* | <0.001* |

*p<0.05 ^a^YBOCS: Yale-Brown Obsessive-compulsive scale; ^b^ IAS: Illness Anxiety Scale; ^c^IES-R: Impact of Event Scale-Revised; ^d^DASS-21: Depression, Anxiety, Stress Scale; ^e^ ISI: Insomnia Severity Index;
